# Supplementary material for: Comparison of RIPASA and ALVARADO scores for risk assessment of acute appendicitis: A systematic review and meta-analysis
Source: PLoS One. 2022 Sep 30;17(9):e0275427. doi: 10.1371/journal.pone.0275427 (PMC9524677; doi:10.1371/journal.pone.0275427)
Supplement: S2 Appendix — (DOCX) [file pone.0275427.s002.docx]

**Additional File 2. QUADAS checklist**

|  | Risk of bias | | | | Applicability concerns | | |
| --- | --- | --- | --- | --- | --- | --- | --- |
| **First author and publication year** | **Patient selection** | **Index Test** | **Reference standard** | **Flow and timing** | **Patient selection** | **Index Test** | **Reference standard** |
| **Sherhryar et al. 2020** | 2 | 2 | 2 | 2 | 2 | 2 | 1 |
| **Dezfuli et al. 2020** | 2 | 2 | 2 | 2 | 2 | 2 | 1 |
| **Korkut et al. 2020** | 2 | 1 | 2 | 2 | 2 | 2 | 1 |
| **Şenocak et al. 2020** | 2 | 2 | 2 | 2 | 3 | 1 | 1 |
| **Devarajan et al. 2019** | 2 | 1 | 3 | 2 | 2 | 1 | 2 |
| **Ozdemir et al. 2019** | 2 | 2 | 2 | 1 | 2 | 1 | 2 |
| **Rohat et al. 2019** | 2 | 3 | 2 | 2 | 1 | 1 | 2 |
| **Akbar et al. 2019** | 1 | 2 | 2 | 1 | 1 | 1 | 1 |
| **Bolìvar-Rodriguez et al. 2018** | 2 | 2 | 2 | 2 | 2 | 1 | 1 |
| **Ansara et al. 2018** | 2 | 2 | 3 | 2 | 2 | 3 | 2 |
| **Patil et al. 2018** | 2 | 3 | 2 | 3 | 3 | 1 | 2 |
| **Chavan et al. 2018** | 1 | 2 | 2 | 2 | 1 | 2 | 3 |
| **Abdelrhman et al. 2018** | 2 | 3 | 1 | 1 | 2 | 3 | 2 |
| **Pasumarthi et al. 2018** | 3 | 2 | 2 | 1 | 2 | 1 | 1 |
| **Elhosseiny et al. 2018** | 2 | 2 | 2 | 1 | 1 | 2 | 2 |
| **Nancharaiah et al. 2018** | 2 | 2 | 2 | 1 | 1 | 2 | 2 |
| **Arroyo-Rangel et al. 2017** | 3 | 2 | 2 | 2 | 2 | 2 | 3 |
| **Rodrigues et al. 2017** | 2 | 1 | 2 | 1 | 2 | 2 | 1 |
| **Karami et al. 2017** | 2 | 1 | 2 | 2 | 3 | 1 | 1 |
| **Chae et al. 2017** | 1 | 2 | 2 | 2 | 1 | 1 | 1 |
| **Regar et al. 2017** | 2 | 2 | 2 | 3 | 1 | 2 | 2 |
| **Subramani et al.** | 2 | 2 | 3 | 2 | 1 | 2 | 2 |
| **Sean K Golden et al. 2016** | 2 | 2 | 2 | 2 | 1 | 3 | 2 |
| **Muduli et al. 2016** | 2 | 3 | 2 | 2 | 2 | 1 | 1 |
| **Sinnet et al. 2016** | 2 | 2 | 2 | 2 | 3 | 2 | 2 |
| **Liu et al. 2015** | 3 | 2 | 3 | 2 | 3 | 2 | 2 |
| **Srikantaiah et al. 2015** | 2 | 2 | 2 | 2 | 1 | 1 | 2 |
| **Verma et al. 2015** | 2 | 1 | 2 | 1 | 1 | 1 | 2 |
| **Walczak et al. 2015** | 2 | 1 | 2 | 1 | 1 | 2 | 2 |
| **NaNjuNdaiah et al. 2014** | 3 | 1 | 2 | 2 | 2 | 2 | 1 |
| **Erdem et al. 2013** | 2 | 2 | 3 | 2 | 2 | 2 | 1 |
| **Alnjadat et al.** | 1 | 1 | 1 | 2 | 1 | 2 | 2 |
| **Chong et al.2011** | 2 | 1 | 1 | 2 | 1 | 2 | 2 |
