# Supplementary material for: Comparison of RIPASA and ALVARADO scores for risk assessment of acute appendicitis: A systematic review and meta-analysis
Source: PLoS One. 2022 Sep 30;17(9):e0275427. doi: 10.1371/journal.pone.0275427 (PMC9524677; doi:10.1371/journal.pone.0275427)
Supplement: Attachment — Submitted filename: PONE-D-22-21559.docx [file pone.0275427.s003.docx]

PLOS ONE

COMPARISON OF RIPASA AND ALVARADO SCORES FOR RISK ASSESSMENT OF ACUTE APPENDICITIS: A SYSTEMATIC REVIEW AND META-ANALYSIS

--Manuscript Draft--

| Manuscript Number: | PONE-D-22-21559 |
| --- | --- |
| Article Type: | Research Article |
| Full Title: | COMPARISON OF RIPASA AND ALVARADO SCORES FOR RISK ASSESSMENT OF ACUTE APPENDICITIS: A SYSTEMATIC REVIEW AND META-ANALYSIS |
| Short Title: | RIPASA AND ALVARADO SCORES FOR RISK ASSESSMENT OF ACUTE APPENDICITIS: A META-ANALYSIS |
| Corresponding Author: | Antonella Agodi  University of Catania Catania, ITALY |
| Keywords: | Appendicitis; Alvarado; RIPASA; Clinical score; risk; diagnosis; risk management |
| Abstract: | Background  In the last decades, several clinical scores have been developed and currently used to improve the diagnosis and risk management of patients with suspected acute appendicitis (AA). However, some of them exhibited different values of sensitivity and specificity. Here, we conducted a systematic review and metanalysis of epidemiological studies, which compared RIPASA and Alvarado scores for the diagnosis of AA.  Methods  The current systematic review was conducted using PubMed and Web of Sciences databases. Selected studies had to compare RIPASA and Alvarado scores on patients with suspected AA and to report diagnostic parameters. Summary estimates of sensitivity and specificity were calculated by the Hierarchical Summary Receiver Operating Curve (HSROC) using STATA 17 (STATA Corp, College Station, TX) and MetaDiSc (version 1.4) software.  Results  We included a total of 33 articles, reporting data from 35 studies. For the Alvarado score, the Hierarchical Summary Receiver Operating Curve (HSROC) model produced a summary sensitivity of 0.72 (95%CI = 0.66 - 0.77), and a summary specificity of 0.77 (95%CI = 0.70 – 0.82). For the RIPASA score, the HSROC model produced a summary sensitivity of 0.95 (95%CI = 0.92 - 0.97), and a summary specificity of 0.71 (95%CI = 0.60 – 0.80).  Conclusion  Our work points out benefits and drawbacks of the two scoring systems used for the diagnosis of AA. Results reveal that the RIPASA score has higher sensitivity but lower specificity than the Alvarado score. Since these scoring systems showed different sensitivity and specificity parameters, it is still necessary to develop novel scores for the risk assessment of patients with suspected AA. |
| Order of Authors: | Giuliana Favara |
|  | Andrea Maugeri |
|  | Martina Barchitta |
|  | Andrea Ventura |
|  | Guido Basile |
|  | Antonella Agodi |
| Additional Information: |  |
| Question | Response |
| Financial Disclosure | The authors received no specific funding for this work. |

| Enter a financial disclosure statement that describes the sources of funding for the work included in this submission. Review the [submission guidelines](http://journals.plos.org/plosone/s/submission-guidelines#loc-financial-disclosure-statement) for detailed requirements. View published research articles from [PLOS ONE](http://journals.plos.org/plosone/) for specific examples.  This statement is required for submission and will appear in the published article if the submission is accepted. Please make sure it is accurate. |  |
| --- | --- |
| Unfunded studies Enter: The author(s) received no specific funding for this work. Funded studies Enter a statement with the following details: - Initials of the authors who received each award - Grant numbers awarded to each author - The full name of each funder - URL of each funder website - Did the sponsors or funders play any role in the study design, data collection and analysis, decision to publish, or preparation of the manuscript? - NO - Include this sentence at the end of your statement: The funders had no role in study design, data collection and analysis, decision to publish, or preparation of the manuscript. - YES - Specify the role(s) played. |  |
| * typeset |  |
| Competing Interests  Use the instructions below to enter a competing interest statement for this submission. On behalf of all authors, disclose any [competing interests](http://journals.plos.org/plosone/s/competing-interests) that could be perceived to bias this work—acknowledging all financial support and any other relevant financial or nonfinancial competing interests.  This statement is required for submission and will appear in the published article if the submission is accepted. Please make | The authors have declared that no competing interests exist |

| sure it is accurate and that any funding sources listed in your Funding Information later in the submission form are also declared in your Financial Disclosure statement.  View published research articles from [PLOS ONE](http://journals.plos.org/plosone/) for specific examples. |  |
| --- | --- |
| NO authors have competing interests Enter: The authors have declared that no competing interests exist. Authors with competing interests Enter competing interest details beginning with this statement: I have read the journal's policy and the authors of this manuscript have the following competing interests: [insert competing interests here] |  |
| * typeset |  |
| Ethics Statement  Enter an ethics statement for this submission. This statement is required if the study involved:   - Human participants - Human specimens or tissue - Vertebrate animals or cephalopods - Vertebrate embryos or tissues - Field research   Write "N/A" if the submission does not require an ethics statement.  General guidance is provided below. Consult the [submission guidelines](http://journals.plos.org/plosone/s/submission-guidelines#loc-guidelines-for-specific-study-types) for detailed instructions. Make sure that all information entered here is included in the Methods section of the manuscript. | N/A |

Format for specific study types

Human Subject Research (involving human

participants and/or tissue)

Give the name of the institutional review

board or ethics committee that approved the

study

•

Include the approval number and/or a

statement indicating approval of this

research

•

Indicate the form of consent obtained

(

written/oral) or the reason that consent was

not obtained (e.g. the data were analyzed

anonymously)

•

Animal Research (involving vertebrate

animals, embryos or tissues)

Provide the name of the Institutional Animal

Care and Use Committee (IACUC) or other

relevant ethics board that reviewed the

study protocol, and indicate whether they

approved this research or granted a formal

waiver of ethical approval

•

Include an approval number if one was

obtained

•

If the study involved

non-human primates

,

add

additional details

about animal welfare

and steps taken to ameliorate suffering

•

If anesthesia, euthanasia, or any kind of

animal sacrifice is part of the study, include

briefly which substances and/or methods

were applied

•

Field Research

Include the following details if this study

involves the collection of plant, animal, or

other materials from a natural setting:

Field permit number

•

Name of the institution or relevant body that

granted permission

•

Data Availability

Authors are required to make all data

underlying the findings described fully

available, without restriction, and from the

time of publication. PLOS allows rare

exceptions to address legal and ethical

concerns. See the

[PLOS Data Polic](http://journals.plos.org/plosone/s/data-availability)

[y](http://journals.plos.org/plosone/s/data-availability)

and

[FA](http://journals.plos.org/plosone/s/data-availability#loc-faqs-for-data-policy)

[Q](http://journals.plos.org/plosone/s/data-availability#loc-faqs-for-data-policy)

for detailed information.

Yes - all data are fully available without restriction

| A Data Availability Statement describing where the data can be found is required at submission. Your answers to this question constitute the Data Availability Statement and will be published in the article, if accepted. |  |
| --- | --- |
| Important: Stating ‘data available on request from the author’ is not sufficient. If your data are only available upon request, select ‘No’ for the first question and explain your exceptional situation in the text box. |  |
| Do the authors confirm that all data underlying the findings described in their manuscript are fully available without restriction? |  |
| Describe where the data may be found in full sentences. If you are copying our sample text, replace any instances of XXX with the appropriate details.   - If the data are held or will be held in a public repository, include URLs, accession numbers or DOIs. If this information will only be available after acceptance, indicate this by ticking the box below. For example: All XXX files   are available from the XXX database (accession number(s) XXX, XXX.).   - If the data are all contained within the manuscript and/or Supporting Information files, enter the following: All relevant data are within the manuscript and its Supporting Information files. - If neither of these applies but you are able to provide details of access elsewhere, with or without limitations, please do so. For example:   Data cannot be shared publicly because of [XXX]. Data are available from the XXX Institutional Data Access / Ethics Committee (contact via XXX) for researchers who meet the criteria for access to confidential data.  The data underlying the results presented in the study are available from (include the name of the third party | All relevant data are within the manuscript and its Supporting Information files |

| and contact information or URL).  • This text is appropriate if the data are owned by a third party and authors do not have permission to share the data.  * typeset |  |
| --- | --- |
| Additional data availability information: |  |

Cover Letter	[Click here to access/download;Manuscript;Draft_PlosOne.docx](https://www.editorialmanager.com/pone/download.aspx?id=31598022&guid=dc3e3d5f-30f3-4b51-ad37-c5289bd54b72&scheme=1)

1	**COMPARISON OF RIPASA AND ALVARADO SCORES FOR RISK ASSESSMENT OF**

# 2 ACUTE APPENDICITIS: A SYSTEMATIC REVIEW AND META-ANALYSIS

3

4

5	**Favara Giuliana^1^, Maugeri Andrea^1^, Barchitta Martina^1^, Ventura Andrea^2^, Basile Guido^2^,**

# 6 Agodi Antonella^1, *^

7

8

9 ^1^ Department of Medical and Surgical Sciences and Advanced Technologies "GF Ingrassia", 10 University of Catania, Via S. Sofia 87, 95123, Catania, Italy.

11 ^2^ Department of General Surgery and Medical-Surgical Specialties, University of Catania, via S.
12 Sofia, 78, 95123, Catania, Italy.

13

14

15

# 16 * Corresponding author: agodia@unict.it

17

18

19

20

21	**Short title: RIPASA AND ALVARADO SCORES FOR RISK ASSESSMENT OF ACUTE**

# 22 APPENDICITIS: A META-ANALYSIS

23

24

25

26

**Abstract**

# Background

In the last decades, several clinical scores have been developed and currently used to improve the diagnosis and risk management of patients with suspected acute appendicitis (AA). However, some of them exhibited different values of sensitivity and specificity. Here, we conducted a systematic review and metanalysis of epidemiological studies, which compared RIPASA and Alvarado scores for the diagnosis of AA.

# Methods

The current systematic review was conducted using PubMed and Web of Sciences databases. Selected studies had to compare RIPASA and Alvarado scores on patients with suspected AA and to report diagnostic parameters. Summary estimates of sensitivity and specificity were calculated by the

Hierarchical Summary Receiver Operating Curve (HSROC) using STATA 17 (STATA Corp, College Station, TX) and MetaDiSc (version 1.4) software.

# Results

We included a total of 33 articles, reporting data from 35 studies. For the Alvarado score, the Hierarchical Summary Receiver Operating Curve (HSROC) model produced a summary sensitivity of 0.72 (95%CI = 0.66 - 0.77), and a summary specificity of 0.77 (95%CI = 0.70 – 0.82). For the RIPASA score, the HSROC model produced a summary sensitivity of 0.95 (95%CI = 0.92 - 0.97), and a summary specificity of 0.71 (95%CI = 0.60 – 0.80).

# Conclusion

Our work points out benefits and drawbacks of the two scoring systems used for the diagnosis of AA. Results reveal that the RIPASA score has higher sensitivity but lower specificity than the Alvarado score. Since these scoring systems showed different sensitivity and specificity parameters, it is still necessary to develop novel scores for the risk assessment of patients with suspected AA.

**Keywords:** Appendicitis; Alvarado; RIPASA; Clinical score; risk; diagnosis; risk management **Introduction**

Acute appendicitis (AA) represents one of the most frequent disorders in abdominal surgery, characterizing patients admitted to the emergency department, with a prevalence ranging from 7 to 12 % in the general population [1, 2]. If untreated or undiagnosed, AA could lead to a higher risk of adverse outcomes, including mortality. Despite its common occurrence, the diagnosis of AA is still challenging for clinicians, suggesting the need of novel approaches to improve patients’ management [3, 4]. Indeed, clinical presentation of AA is commonly atypical and easily mistaken for other conditions, with only about 40% of the cases presenting typical signs and symptoms (i.e. periumbilical pain, nausea, vomiting, pain migration to the right lower quadrant) [5-7].

In the last decades, several scoring systems have been developed to assist clinicians in the assessment of patients with suspected appendicitis [8, 9]. Among these, the ALVARADO score - proposed for the first time in 1986 - is one of the most widely used in the diagnosis of AA based on 6 clinical parameters and 2 laboratory measurements (i.e. localized tenderness in the right lower quadrant, leucocytosis, migration of pain, leukocyte shift to the left, temperature elevation, nausea-vomiting, anorexia and rebound pain) [8]. Despite not being specific enough, a score of 4–5 is compatible with the diagnosis of AA, a score of 7-8 indicates a probable appendicitis, and a score of 9-10 indicates a very probable AA [10, 11]. However, the Alvarado score is also considered lacking of some parameters, including age, gender and duration of symptoms, which have shown to be crucial in the diagnosis of AA [3, 12]. The RIPASA is one of the most recently developed scoring systems, which is based on six additional clinical and personal patients’ parameters than those of Alvarado score. In this case, a RIPASA score of more than 7.5 is considered positive for appendicitis [1, 8, 11, 13-15]. Although RIPASA and Alvarado scores are the most commonly used in clinical practice, no clear indication exists for choosing what scoring system might be more suitable for patients at risk of AA

[16]. Here, we conducted a systematic review and metanalysis of epidemiological studies comparing RIPASA and Alvarado scores, in order to identify which is the one providing more accurate diagnosis of AA.

# Material and Methods

## Literature search and selection criteria

The current systematic review was conducted in accordance with the Preferred Reporting Items for

Systematic Reviews and Meta-analyses (PRISMA) statements and the Cochrane Handbook’s guidelines (PRISMA checklist available in **S1 Appendix**) [17]. Two authors (GB and AV) conducted a literature search of articles, using the databases PubMed and Web of Sciences. The electronic search strategy included the following keywords: ((Appendicitis) AND (RIPASA) AND (Alvarado)). The last search was done on 21 July 2021. After identifying and removing duplicates, two authors also conducted a cross-search through the articles cited by the studies, aiming to identify additional articles to be included in the systematic review. Selected studies had to meet the following inclusion criteria: (i) observational studies; (ii) which provided full-text and written in English language; (iii) which included patients with suspected acute appendicitis (iv) and compared RIPASA and Alvarado scores. By contrast, the following articles were excluded: (i) experimental studies; (ii) studies conducted only on a specific population (e.g. pregnant women or paediatric patients); (iii) studies not comparing the two above-mentioned scoring systems; (iv) studies conducted on patients with an already established cause of abdominal pain and/or patients who experienced pain for a prolonged period; (v) letters, comments, case reports, case series, reviews.

Titles and abstracts of all identified articles were independently screened by two authors (GB and AV). Articles potentially eligible were full-text reviewed to assess whether eligibility criteria were fully met. Discordant opinions between investigators were resolved by consulting a third author (AA).

## Data extraction

The following information was extracted from all included studies: first author, year of publication, study design, sample size, age, sex, histologically confirmed acute appendicitis, other previous diagnoses, computerized tomography (CT) performed. In addition, for both the RIPASA and Alvarado scores, the authors collected the following information: specificity, sensitivity, positive predictive value, negative predictive value, diagnostic accuracy, negative appendicectomy rate, area under the roc-curve, positive likelihood ratio, negative likelihood ratio. Discordant opinions between investigators were resolved by consulting a third author (AA).

## Definitions of RIPASA and ALVARADO scores

Clinical Scoring Systems are useful to group patients according to their symptoms and signs, and to identify patients with suspected appendicitis. Alvarado clinical score includes 6 clinical parameters and 2 laboratory measurements, which are relevant in the diagnosis of acute appendicitis. Among these, migration of abdominal pain to the right iliac fossa, anorexia or ketones in the urine, nausea or vomiting, localized tenderness in the right iliac fossa, rebound pain, body temperature more than 37.3 °C, leucocytosis, and neutrophilia. Alvarado score indicates a confirmed, probable or very probable diagnosis of acute appendicitis, in the case of a score of 4-6, 7-8, or 9-10, respectively. Commonly, a score of 7.0 is considered as positive for appendicitis [10, 11].

RIPASA clinical score includes the following 15 parameters: age, gender, right iliac fossa pain, migration of pain to the right iliac fossa, nausea or vomiting, anorexia, duration of symptoms, localized tenderness in the right iliac fossa, guarding, rebound tenderness, Rovsing' s sign, fever, raised white cell count, negative urinalysis and foreign national registration identity card. Commonly, a score above 7.5 is considered as positive for the diagnosis of appendicitis [1, 8, 11, 13-15].

## Risk of bias and quality assessment

The methodological quality of the included studies was assessed using a set of criteria for the Quality Assessment of Diagnostic Accuracy Studies (QUADAS-2). By considering 4 domains (i.e., patient selection, index test, reference standard, and flow and timing), this approach is useful for the evaluation of diagnostic accuracy studies. In particular, the questions can be answered using “low”, “high” or “unclear” to judge the risk of bias [18].

## Statistical analysis

Meta-analysis of diagnostic test accuracy requires a statistically rigorous approach based on hierarchical models that respect the binomial data structure. In the present study, we first obtained for each score the forest plots of sensitivity and specificity and their 95% Confidence Intervals (CI) based on a random-effects model and using the MetaDiSc software (version 1.4). The heterogeneity was assessed with the I^2^ statistic. Next, the summary estimates of sensitivity and specificity were calculated by the Hierarchical Summary Receiver Operating Curve (HSROC), using the package

Metandi for STATA 17 statistical software (STATA Corp, College Station, TX). To visualize the HSROC curve, we also used the command metandiplot.

# Results

## Selection and characteristics of included studies

**Figure 1** reported the PRISMA flow diagram describing the study selection process. A total of 75 studies were identified from the literature search, of which 53 were screened after removing duplicates. After full-text screening of 31 articles deemed eligible for research purposes, 2 studies not comparing two scoring systems considered, 2 reviews, and 1 study not written in English were excluded. After a cross-search through the articles cited by the studies, the authors identified 7 additional articles to be included. Hence, a total of 33 articles, describing data from 35 studies, were included in the present systematic review and meta-analysis. **Table 1** shows the main characteristics

(i.e., country, type of study, sample size) of the included studies, as well as characteristics of patients (i.e., age, sex). **Table 2**, instead, summarizes statistical parameters of RIPASA and ALVARADO scores, respectively.

## Main characteristics of included studies

All the included studies were published between 2011 and 2020. In particular, most of the studies were conducted in South-Eastern countries, of which 14 in India, 5 in Turkey, 2 in Pakistan, 2 in Egypt, 2 in Iran, 1 in Jordan, 1 in China, 1 in Korea, 1 in Brunei, 2 in Mexico, 1 in USA and 1 in Poland. With respect to the study design, 30 were observational studies with prospective (n=26) or retrospective (n=4) data collection, and 3 cross-sectional. The overall sample size ranged from 56 to 600 participants, whose age ranged from 25 to 30 years. Although gender distribution throughout the studies was fairly balanced, almost all studies reported a higher proportion of men. The most commonly considered symptom to identify patients with AA was the pain in Right Iliac Fossa. Moreover, some studies required more extensive list of clinical symptoms, as well as advanced imaging techniques.

## Cut-offs of scoring systems

In the various studies, diagnostic parameters for RIPASA and Alvarado scores were calculated according to different cut-offs. Most of studies used 7.0 and 7.5 as conventional cut-offs for Alvarado and RIPASA scores, respectively. Accordingly, patients were considered as affected by AA if their scores exceeded these cut-off values. However, Korkut et al. and Ozdemir et al. used the value of 8 for the Alvarado, and the values of 10 or 12 for the RIPASA, respectively. Reasons of using different cut-offs may be explained by the aim to improve the diagnostic parameters of the scores. For all the studies considered, the gold standard is given by the histopathological exam performed post-surgery.

## Scoring systems performances

Overall, the present systematic review included 5384 patients with AA who were tested with the RIPASA and Alvarado scores. The sensitivity values ranged from 16.4% to 100% for the RIPASA score, and from 14.8% to 97.2% for the Alvarado score (**Fig 2**). Interestingly, all studies reported a higher sensitivity for the RIPASA score than for the Alvarado score. Most of the studies reported higher values of specificity for the Alvarado score than for the RIPASA score. The specificity values ranged from 9% to 100% for the RIPASA score, and from 16% to 100% for the Alvarado score (**Fig 3**). The majority of studies reported higher Positive Predictive Value for the Alvarado score. Conversely, the majority of studies reported higher Negative Predictive Values for the RIPASA score. Moreover, in the studies included in the present meta-analysis, the RIPASA score showed higher values of diagnostic accuracy and AUC compared to the Alvarado.

**Figure 4** shows hierarchical summary estimates of sensitivity and specificity for the Alvarado and the RIPASA scores, respectively. The graphs also report a 95% prediction ellipse for the individual values of sensitivity and specificity, and the 95% confidence ellipse around the mean values of sensitivity and specificity. For the Alvarado score (**Fig 4A**), the HSROC model produced a summary sensitivity of 0.72 (95%CI = 0.66 - 0.77), and a summary specificity of 0.77 (95%CI = 0.70 – 0.82). The heterogeneity was I^2^ = 0.90 for the sensitivity and I^2^ = 0.59 for the specificity. For the RIPASA score (**Fig 4B**), the HSROC model produced a summary sensitivity of 0.95 (95%CI = 0.92 - 0.97), and a summary specificity of 0.71 (95%CI = 0.60 – 0.80). The heterogeneity was I^2^ = 0.76 for the sensitivity and I^2^ = 0.70 for the specificity.

## Quality assessment

The details of the quality assessment are reported in **S2 Appendix**. In general, the risk of bias was unclear or high for all domains under investigation (i.e., patient selection, index test, reference standard, and flow and timing). Similarly, we noted unclear or high concerns of applicability for all studies.

**Fig 1.** Prisma flow diagram describing study selection process

**Fig 2.** Plots of individual values of sensitivity for the Alvarado (A) and RIPASA (B) scores

**Fig 3.** Plots of individual values of specificity for the Alvarado (A) and RIPASA (B) scores

**Fig 4.** The HSROC of sensitivity and specificity for the Alvarado (A) and RIPASA (B) scores

# Discussion

AA is one of the most common causes of acute abdominal pain, posing a pressing issue for general surgeons due to its clinical variability and high prevalence [3]. Although a wide range of diagnostic tests, such as CT scan and ultrasonography, hold great promise in clinical practice, identifying a normal appendix is still challenging both for avoiding unnecessary surgical intervention and reducing assistance costs [19, 20]. Moreover, complications related to the inflammation of the appendix further complicate patient's prognosis, also suggesting the need of implementing prediction scoring systems [20]. In this scenario, the use of clinical scoring systems can help healthcare providers in improving decision-making, patients’ management and identification of suspected appendicitis [3]. Moreover, several lines of evidence suggest that the integrated use of clinical scoring systems and diagnostics images allow to correctly identify patients with AA [3, 8]. Among the most common scores, RIPASA and Alvarado constitute the most utilized to clinically diagnose appendicitis in suspected patients [21]. In this scenario, we carried out a systematic review and meta-analysis of epidemiological studies comparing these two scores in terms of sensitivity and specificity. In line with previous evidence, our results reveal that the RIPASA score has higher sensitivity but lower specificity than the Alvarado score. To our knowledge, the strength of our work was represented by the lack of systematic reviews and meta-analyses in medical literature published on the same topic. Moreover, our study considered two scoring systems that have the advantages of being easy to use for clinicians, also requiring low healthcare costs to be applied. However, our study had some limitations to be considered. Firstly, most studies included in the present meta-analysis considered different cut-off values for the RIPASA and Alvarado scoring systems. Therefore, this could provide a distortion in terms of statistical parameters, also representing a source of bias. Secondly, these scoring systems are mainly based on patient’ clinical parameters measured in emergency situations and critical environments, which in turn could lead to wrong diagnoses and scoring systems calculation. Moreover, using these two scores could make difficult the diagnosis of AA for specific subgroups of patients, including those with older age, diabetes mellitus and paediatric patients. Thirdly, most of the studies included in the present meta-analysis did not compare RIPASA and Alvarado scores with other diagnostic tests used in clinical practice. With these considerations in mind, the present systematic review and meta-analysis points out benefits and drawbacks of the two widely used scoring systems for the diagnosis of AA. Specifically, we found that the RIPASA scoring system can be useful both for excluding the diagnosis of AA and for relaying intermediate-risk patients to more accurate diagnostic imaging techniques.

However, further studies are needed to identify future strategies – also implementing the existing scores and/or developing innovative tools – in order to produce more accurate results and to improve the risk assessment in the clinical practice.

# Conclusions

In conclusion, the early diagnosis of patients with suspected AA is still a challenge for clinical practitioners and public health professionals. Although the existing scoring systems help in the risk assessment and in the prediction of clinical deterioration, these scores show variable values of specificity and sensitivity. In our study, the RIPASA score had a good performance in identifying true positive patients, while the Alvarado score was better in predicting true negative patients. For this reason, further research should be encouraged to develop novel scores and strategies for improving the risk assessment of patients with suspected AA.

# Supporting Information

**S1 Appendix.** PRISMA checklist

**S2 Appendix.** Quality assessment of the included studies

**Competing interests**

The authors declared no conflict of interest.

**Funding**

The authors received no funding.

# Author contributions

Protocol development: GB and AA; data collection: AV and GB. Data analysis: GF, AM and MB.

Manuscript writing: GF and AM. Manuscript editing: all the authors.

## **6. References**

1. Noor S, Wahab A, Afridi G, Ullah K. Comparing Ripasa Score And Alvarado Score In An

Accurate Diagnosis Of Acute Appendicitis. J Ayub Med Coll Abbottabad. 2020;32(1):38-41. PubMed PMID: 32468752.

2. Krzyzak M, Mulrooney SM. Acute Appendicitis Review: Background, Epidemiology, Diagnosis, and Treatment. Cureus. 2020;12(6):e8562. Epub 20200611. doi: 10.7759/cureus.8562.

PubMed PMID: 32670699; PubMed Central PMCID: PMCPMC7358958.

3. Di Saverio S, Podda M, De Simone B, Ceresoli M, Augustin G, Gori A, et al. Diagnosis and treatment of acute appendicitis: 2020 update of the WSES Jerusalem guidelines. World J Emerg Surg.

2020;15(1):27. Epub 20200415. doi: 10.1186/s13017-020-00306-3. PubMed PMID: 32295644; PubMed Central PMCID: PMCPMC7386163.

4. Humes	DJ,	Simpson	J.	Acute	appendicitis.	BMJ.	2006;333(7567):530-4.	doi:

10.1136/bmj.38940.664363.AE.	PubMed	PMID:	16960208;	PubMed	Central	PMCID: PMCPMC1562475.

5. Golden SK, Harringa JB, Pickhardt PJ, Ebinger A, Svenson JE, Zhao YQ, et al. Prospective evaluation of the ability of clinical scoring systems and physician-determined likelihood of appendicitis to obviate the need for CT. Emerg Med J. 2016;33(7):458-64. Epub 20160302. doi:

10.1136/emermed-2015-205301.	PubMed	PMID:	26935714;	PubMed	Central	PMCID:

PMCPMC5443621.

6. Snyder MJ, Guthrie M, Cagle S. Acute Appendicitis: Efficient Diagnosis and Management. Am Fam Physician. 2018;98(1):25-33. PubMed PMID: 30215950.
7. Podda M, Pisanu A, Sartelli M, Coccolini F, Damaskos D, Augustin G, et al. Diagnosis of acute appendicitis based on clinical scores: is it a myth or reality? Acta Biomed.

2021;92(4):e2021231. Epub 20210902. doi: 10.23750/abm.v92i4.11666. PubMed PMID: 34487066; PubMed Central PMCID: PMCPMC8477120.

8. Frountzas M, Stergios K, Kopsini D, Schizas D, Kontzoglou K, Toutouzas K. Alvarado or

RIPASA score for diagnosis of acute appendicitis? A meta-analysis of randomized trials. Int J Surg. 2018;56:307-14. Epub 20180712. doi: 10.1016/j.ijsu.2018.07.003. PubMed PMID: 30017607.

9. Şenocak R, Kaymak Ş. Diagnostic accuracy of ultrasonography and scoring systems: The effects on the negative appendectomy rate and gender. Ulus Travma Acil Cerrahi Derg. 2020;26(2):306-13. doi: 10.14744/tjtes.2019.86717. PubMed PMID: 32185777.
10. Dezfuli SAT, Yazdani R, Khorasani M, Hosseinikhah SA. Comparison between the specificity and sensitivity of the RIPASA and Alvarado Scoring systems in the diagnosis of acute appendicitis among patients with complaints of right iliac fossa. AIMS Public Health. 2020;7(1):1-9. Epub 20200102. doi: 10.3934/publichealth.2020001. PubMed PMID: 32258184; PubMed Central PMCID: PMCPMC7109537.
11. Akbar I, Shehzad JA, Ali S. Diagnostic Accuracy Of Ripasa Score. J Ayub Med Coll Abbottabad. 2019;31(3):411-4. PubMed PMID: 31535517.
12. Moris D, Paulson EK, Pappas TN. Diagnosis and Management of Acute Appendicitis in

Adults: A Review. JAMA. 2021;326(22):2299-311. doi: 10.1001/jama.2021.20502. PubMed PMID: 34905026.

13. Chong CF, Adi MI, Thien A, Suyoi A, Mackie AJ, Tin AS, et al. Development of the RIPASA score: a new appendicitis scoring system for the diagnosis of acute appendicitis. Singapore Med J. 2010;51(3):220-5. PubMed PMID: 20428744.
14. Chisthi MM, Surendran A, Narayanan JT. RIPASA and air scoring systems are superior to alvarado scoring in acute appendicitis: Diagnostic accuracy study. Ann Med Surg (Lond).

2020;59:138-42. Epub 20200924. doi: 10.1016/j.amsu.2020.09.029. PubMed PMID: 33024555; PubMed Central PMCID: PMCPMC7527659.

15. Malik MU, Connelly TM, Awan F, Pretorius F, Fiuza-Castineira C, El Faedy O, et al. The

RIPASA score is sensitive and specific for the diagnosis of acute appendicitis in a western population. Int J Colorectal Dis. 2017;32(4):491-7. Epub 20161215. doi: 10.1007/s00384-016-2713-4. PubMed PMID: 27981378.

16. Mantoglu B, Gonullu E, Akdeniz Y, Yigit M, Firat N, Akin E, et al. Which appendicitis scoring system is most suitable for pregnant patients? A comparison of nine different systems. World J Emerg Surg. 2020;15(1):34. Epub 20200518. doi: 10.1186/s13017-020-00310-7. PubMed PMID: 32423408; PubMed Central PMCID: PMCPMC7236497.
17. Page MJ, McKenzie JE, Bossuyt PM, Boutron I, Hoffmann TC, Mulrow CD, et al. The

PRISMA 2020 statement: an updated guideline for reporting systematic reviews. BMJ. 2021;372:n71. Epub 20210329. doi: 10.1136/bmj.n71. PubMed PMID: 33782057; PubMed Central PMCID: PMCPMC8005924.

18. Whiting PF, Rutjes AW, Westwood ME, Mallett S, Deeks JJ, Reitsma JB, et al. QUADAS-2: a revised tool for the quality assessment of diagnostic accuracy studies. Ann Intern Med. 2011;155(8):529-36. doi: 10.7326/0003-4819-155-8-201110180-00009. PubMed PMID: 22007046.
19. Pinto F, Pinto A, Russo A, Coppolino F, Bracale R, Fonio P, et al. Accuracy of

ultrasonography in the diagnosis of acute appendicitis in adult patients: review of the literature. Crit Ultrasound J. 2013;5 Suppl 1:S2. Epub 20130715. doi: 10.1186/2036-7902-5-S1-S2. PubMed PMID: 23902717; PubMed Central PMCID: PMCPMC3711731.

20. Ozkan S, Duman A, Durukan P, Yildirim A, Ozbakan O. The accuracy rate of Alvarado score, ultrasonography, and computerized tomography scan in the diagnosis of acute appendicitis in our center. Niger J Clin Pract. 2014;17(4):413-8. doi: 10.4103/1119-3077.134001. PubMed PMID: 24909462.
21. Kularatna M, Lauti M, Haran C, MacFater W, Sheikh L, Huang Y, et al. Clinical Prediction Rules for Appendicitis in Adults: Which Is Best? World J Surg. 2017;41(7):1769-81. doi:

10.1007/s00268-017-3926-6. PubMed PMID: 28258458.

22. Korkut M, Bedel C, Karancı Y, Avcı A, Duyan M. Accuracy of Alvarado, Eskelinen,

Ohmann, RIPASA and Tzanakis Scores in Diagnosis of Acute Appendicitis; a Cross-sectional Study. Arch Acad Emerg Med. 2020;8(1):e20. Epub 20200313. PubMed PMID: 32259117; PubMed Central PMCID: PMCPMC7130437.

23. Devarajan L, Ramesh A, Gayathri S. A prospective study of comparison of RIPASA and Alvarado scores for the diagnosis of acute appendicitis in government Vellore medical college. J.

Evolution Med. Dent. Sci. 2019;8(19):1578- 1581, DOI: 10.14260/jemds/2019/349

24. Ozdemir U Z, Ozdemir H, Sunamak O, Akyuz C, Torun M. “Comparison of the reliability of scoring systems in the light of histopathological results in the diagnosis of acute appendicitis.” Hong Kong Journal of Emergency Medicine 26 (2018): 323 - 327.
25. Ak R, Doğanay F, Unal Akoğlu E, et al. Predictive value of scoring systems for the diagnosis of acute appendicitis in emergency department patients: Is there an accurate one? *Hong Kong Journal of Emergency Medicine*. 2020;27(5):262-269. doi:[10.1177/1024907919840175](https://doi.org/10.1177/1024907919840175)
26. Bolívar-Rodríguez MA, Osuna-Wong BA, Calderón-Alvarado AB, Matus-Rojas J, DehesaLópez E, Peraza-Garay FJ. [Comparative analysis of diagnostic scales of acute appendicitis:

Alvarado, RIPASA and AIR]. Cir Cir. 2018;86(2):169-74. doi: 10.24875/CIRU.M18000029. PubMed PMID: 29809191.

27. Ansari TS, Kumar VS. Comparison of RIPASA and Alvarado scoring system in acute appendicitis- a descriptive study. J. Evolution Med. Dent. Sci. 2018;7(15):1872-1876, DOI: 10.14260/jemds/2018/422
28. Patil SB, Vishweshvarayya. Validation of RIPASA scoring system for diagnosis of acute appendicitis. J. Evolution Med. Dent. Sci. 2018;7(32):3599-3603, DOI: 10.14260/jemds/2018/808 29. Chavan S, Bhatnagar S, Bendre M, Sinha N. Comparative study of Alvarado score and RIPASA score in the diagnosis of acute appendicitis. Int Surg J 2018;5:1330-4.
30. Abdelrhman T, Al Saeed M, Badr S, Shaban M, Abu-Duruk A, Hatem M. “Validity of RIPASA scoring system as a diagnostic tool of acute appendicitis in comparison with Alvarado scoring system in the Arab population.” International Surgery Journal 5 (2018): 2011.
31. Pasumarthi V, Madhu CP. A comparative study of RIPASA score and ALVARADO score in diagnosis of acute appendicitis. Int Surg J 2018;5:796-801.
32. Elhosseiny, Mostafa M., Eman A. Eltokhy, Omar, Atef Elekiabi, Mostafa. M. Elaidy, Tamer

Youssef Mohamed, El shahidy, A. Mohammed Salem and Ahmed Embaby. “Comparative Study between Alvarado Score& Ripasa Score in Diagnosis of Acute Appendicitis.” Medicine (2018).

33. Nancharaiah P, Aishwarya M, Venkateswarulu M. Comparative Study of Alvarado and RIPASA Scoring System in Diagnosing Acute Appendicitis. Indian J Surg 81, 150–153 (2019).

https://doi.org/10.1007/s12262-018-1752-1

34. Arroyo-Rangel C, Limón IO, Vera Á, Guardiola PM, Sánchez-Valdivieso EA. Sensitivity, Specificity and Reliability of the RIPASA Score for Diagnosis of Acute Appendicitis in Relation to

the Alvarado Score. Cir Esp (Engl Ed). 2018;96(3):149-54. Epub 20180224. doi: 10.1016/j.ciresp.2017.11.013. PubMed PMID: 29486897.

35. Rodrigues W, Sindhu S. “Diagnostic Importance of Alvarado and RIPASA Score in Acute

Appendicitis.” Medicine (2017).

36. Karami MY, Niakan H, Zadebagheri N, Mardani P, Shayan Z, Deilami I. Which One is Better? Comparison of the Acute Inflammatory Response, Raja Isteri Pengiran Anak Saleha Appendicitis and Alvarado Scoring Systems. Ann Coloproctol. 2017;33(6):227-31. Epub 20171231. doi: 10.3393/ac.2017.33.6.227. PubMed PMID: 29354605; PubMed Central PMCID: PMCPMC5768477.
37. Chae MS, Hong CK, Ha YR, Chae MK, Kim YS, Shin TY, et al. Can clinical scoring systems improve the diagnostic accuracy in patients with suspected adult appendicitis and equivocal preoperative computed tomography findings? Clin Exp Emerg Med. 2017;4(4):214-21. Epub

20171020. doi: 10.15441/ceem.16.168. PubMed PMID: 29055962; PubMed Central PMCID: PMCPMC5758620.

38. Regar MK, Choudhary GS, Nogia C, Pipal DK, Agrawal AK, Srivastava H. “Comparison of Alvarado and RIPASA scoring systems in diagnosis of acute appendicitis and correlation with intraoperative and histopathological findings.” International Surgery Journal 4 (2017): 1755-1761.
39. Subramani B, Kalaichelvan L, Selvam G, et al. Comparison between RIPASA and ALVARADO scoring in diagnosing acute appendicitis. J. Evid. Based Med. Healthc. 2017; 4(11), 624-627. DOI: 10.18410/jebmh/2017/121
40. Muduli IC, Rout BK, Mallick SN. Comparison of RIPASA and Alvarado score in diagnosis of acute appendicitis. J. Evolution Med. Dent. Sci. 2016;5(33):1794-1798, DOI: 10.14260/jemds/2016/423
41. Sinnet PR, Chellappa PM, Kumar S, et al. Comparative study on the diagnostic accuracy of the RIPASA score over Alvarado score in the diagnosis of acute appendicitis. J. Evid. Based Med.

Healthc. 2016; 3(80), 4318-4321. DOI: 10.18410/jebmh/2016/920

42. Liu W, Wei Qiang J, Xun Sun R. Comparison of multislice computed tomography and clinical scores for diagnosing acute appendicitis. J Int Med Res. 2015;43(3):341-9. Epub 20150311. doi: 10.1177/0300060514564475. PubMed PMID: 25762518.
43. Srikantaiah HC, Arvind Nayak K. “Validation of RIPASA Scoring System for the Diagnosis of

Acute Appendicitis.” Journal of Evolution of Medical and Dental Sciences 2015; Vol. 4, Issue 100, December 14; Page:16533-16547, DOI: 10.14260/jemds/2015/2461

44. Verma M , Chanchal, Karamveer, Vashist MG, Goyal K, Yadav P. (2016). Comparision of

Alvarado And Ripasa Scoring Systems in Diagnosis of Acute Appendicitis. *Paripex Indian Journal Of Research, 4*.

45. Walczak DA, Pawełczak D, Żółtaszek A, Jaguścik R, Fałek W, Czerwińska M, et al. The

Value of Scoring Systems for the Diagnosis of Acute Appendicitis. Pol Przegl Chir. 2015;87(2):65-

70. doi: 10.1515/pjs-2015-0021. PubMed PMID: 26146097.

46. N N, Mohammed A, Shanbhag V, Ashfaque K, S A P. A Comparative Study of RIPASA Score and ALVARADO Score in the Diagnosis of Acute Appendicitis. J Clin Diagn Res.

2014;8(11):NC03-5. Epub 20141120. doi: 10.7860/JCDR/2014/9055.5170. PubMed PMID: 25584259; PubMed Central PMCID: PMCPMC4290278.

47. Erdem H, Çetinkünar S, Daş K, Reyhan E, Değer C, Aziret M, et al. Alvarado, Eskelinen, Ohhmann and Raja Isteri Pengiran Anak Saleha Appendicitis scores for diagnosis of acute appendicitis. World J Gastroenterol. 2013;19(47):9057-62. doi: 10.3748/wjg.v19.i47.9057. PubMed PMID: 24379631; PubMed Central PMCID: PMCPMC3870559.
48. Alnjadat I, Baha A. “Alvarado versus RIPASA score in diagnosing acute appendicitis.” Rawal Medical Journal 38 (2013): 147-151.
49. Chong CF, Thien A, Mackie AJ, Tin AS, Tripathi S, Ahmad MA, et al. Comparison of RIPASA and Alvarado scores for the diagnosis of acute appendicitis. Singapore Med J.

2011;52(5):340-5. PubMed PMID: 21633767.

**Table 1.** Characteristics of studies included in the systematic review

| **Study** | **Country** | **Study design** | **Sample size** | **Age (years)** | **Sex (% of male)** | **Histologically diagnosis of AA** |
| --- | --- | --- | --- | --- | --- | --- |
| Noor et al. 2020 [1] | Pakistan | Prospective | 300 | 28±10.0 | 58.7% | 270 |
| Dezfuli et al. 2020 [10] | Iran | Prospective | 133 | 28.3 ± 4.8 | 55.6% | 76 |
| Korkut et al. 2020 [22] | Turkey | Prospective | 74 | 36.68 ± 11.97 | 56. 8% | 65 |
| Şenocak et al. 2020 [9] | Turkey | Retrospective | 202 | 25.6±8.8 | 75.2% | 170 |
| Devarajan et al. 2019 [23] | India | Prospective | 250 | NA | 66.0% | 237 |
| Ozdemir et al. 2019 [24] | Turkey | Retrospective | 76 | 33.8 ± 13.2 | 57.9% | 59 |
| Ak et al. 2019 [25] | Turkey | Prospective | 218 | 33 (median) | 48.2% | 107 |
| Akbar et al. 2019 [11] | Pakistan | Prospective | 288 | NA | 57.3% | 252 |
| Bolìvar-Rodriguez et al. 2018 [26] | Mexico | Prospective | 137 | NA | - | 108 |
| Ansara et al. 2018 [27] | India | Prospective | 100 | 32.16 | 57.0% | 74 |
| Patil et al. 2018 [28] | India | Prospective | 150 | NA | 69.3% | NA |
| Chavan et al. 2018 [29] | India | Cross-sectional | 100 | NA | 71.0% | 99 |
| Abdelrhman et al. 2018 [30] | Egypt | Prospective | 100 | NA | 40.0% | 89 |

| Abdelrhman et al. 2018 | Egypt | Prospective | 100 | NA | 59.0% | 82 |
| --- | --- | --- | --- | --- | --- | --- |
| Pasumarthi et al. 2018 [31] | India | Prospective | 116 | 34.4 | 48.3% | 96 |
| Elhosseiny et al. 2018 [32] | Egypt | Cross-sectional | 56 | 28.3 ± 8.1 | 35.7% | 46 |
| Nancharaiah et al. 2018 [33] | India | Prospective | 150 | NA | - | 144 |
| Arroyo-Rangel et al. 2017 [34] | Mexico | Prospective | 100 | 36.5 ± 16.2 | 42.0% | 85 |
| Rodrigues et al. 2017 [35] | India | Prospective | 105 | NA | 45.7% | 86 |
| Karami et al. 2017 [36] | Iran | Prospective | 100 | 32 ± 10 | 66.0% | 88 |
| Chae et al. 2017 [37] | Korea | Retrospective | 189 | NA | 33.3% | 61 |
| Regar et al. 2017 [38] | India | Prospective | 100 | 24.86 | 61.0% | 95 |
| Subramani et al. 2017 [39] | India | Prospective | 96 | 30.58 ±12.3 | 47.9% | 50 |
| Golden et al. 2016 [5] | USA | Prospective | 287 | 31(IQR=12–  88) | 40.1% | NA |
| Muduli et al. 2016 [40] | India | Prospective | 96 | 23.5±9.42 | 72.9% | 73 |
| Sinnet et al. 2016 [41] | India | Cross-sectional | 109 | 28 | 36.7% | 89 |
| Liu et al. 2015 [42] | China | Retrospective | 297 | 47.9 ± 17.6 | 53.2% | 187 |
| Srikantaiah et al. 2015 [43] | India | Prospective | 150 | 25.87 (mean) | 69.3% | 111 |
| Verma et al. 2015 [44] | India | Prospective | 100 | 28.10±10.88 | 67.0% | 91 |
| Walczak et al. 2015 [45] | Poland | Prospective | 94 | 38 (mean) | 51.1% | 59 |
| NaNjuNdaiah et al. 2014 [46] | India | Prospective | 206 | 27.82 ± 9.26 | 61.7% | 184 |
| Erdem et al. 2013 [47] | Turkey | Prospective | 113 | 30.2 ± 10.1 | 54.9% | 77 |
| Erdem et al. 2013 | Turkey | Prospective | 113 | 30.2 ± 10.1 | 54.9% | 77 |
| Alnjadat et al. 2013 [48] | Jordan | Prospective | 600 | 26.52 (mean) | 60.0% | 498 |
| Chong et al.2011 [49] | Brunei | Prospective | 192 | 25.1 ± 12.7 | 47.9% | 101 |

**Table 2.** Characteristics of clinical scoring systems for each study included in the systematic review

| **Study** | **Score** | **Cut – off** | **Sensitivity (%)** | **Specificity (%)** | **PPV (%)** | **NPV %** | **Diagnostic**  **Accuracy**  **(%)** | **Negative appendectomy rate (%)** | **AUC** |
| --- | --- | --- | --- | --- | --- | --- | --- | --- | --- |
| Noor et al. 2020 | Ripasa | 7.5 | 98.5 | 90 | 98.9 | 87.1 | 97.7 | 10 | NA |
| Noor et al. 2020 | Alvarado | 7 | 68.1 | 80 | 96.8 | 21.8 | 69.3 | 20 | NA |
| Dezfuli et al. 2020 | Ripasa | 7.7 | 93.4 | 45.6 | 69.6 | 83.9 | NA | NA | 0.739 |
| Dezfuli et al. 2020 | Alvarado | 6 | 53.9 | 70.2 | 70.7 | 53.3 | NA | NA | 0.662 |
| Korkut et al. 2020 | Ripasa | 12 | 75 | 99.7 | 98.0 | 34.8 | NA | NA | 0.893 |
| Korkut et al. 2020 | Alvarado | 8 | 60.9 | 89.9 | 97.6 | 24.2 | NA | NA | 0.938 |
| Şenocak et al. 2020 | Ripasa | 9.8 | 83.5 | 37.5 | 87.6 | 30 | NA | 12.3 | 0.605 |
| Şenocak et al. 2020 | Alvarado | 7.3 | 75.8 | 65.6 | 92.1 | 33.8 | NA | 7.9 | 0.708 |
| Devarajan et al. 2019 | Ripasa | 7.5 | 98.4 | 90 | 99.5 | 75 | 97 | NA | NA |

| Devarajan et al. 2019 | Alvarado | 7 | 73.7 | 80 | 94.3 | 3.4 | 74 | NA | NA |
| --- | --- | --- | --- | --- | --- | --- | --- | --- | --- |
| Ozdemir et al. 2019 | Ripasa | 10 | 68 | 71 | 89 | 39 | 75 | NA | 0.700 |
| Ozdemir et al. 2019 | Alvarado | 8 | 36 | 82 | 87 | 27 | 56 | NA | 0.600 |
| Ak et al. 2019 | Ripasa | 7.5 | 91.6 | 65.8 | NA | NA | 0.9 | 14.3 | 0.880 |
| Ak et al. 2019 | Alvarado | 5 | 72.9 | 54.1 | NA | NA | 0.7 | 71.4 | 0.710 |
| Akbar et al. 2019 | Ripasa | 7.5 | 98 | 75 | 96.5 | 84.7 | NA | NA | NA |
| Akbar et al. 2019 | Alvarado | 7 | 53 | 75 | NA | NA | NA | NA | NA |
| Bolìvar-Rodriguez et al.  2018 | Ripasa | 7.5 | 97.2 | 27.6 | 83.3 | 72.7 | 82.5 | NA | NA |
| Bolìvar-Rodriguez et al.  2018 | Alvarado | 7 | 97.2 | 27.6 | 83.3 | 72.7 | 82.5 | NA | NA |
| Ansara et al. 2018 | Ripasa | 7.5 | 91.9 | 80.8 | 93.2 | 77.8 | 89 | 6.8 | NA |
| Ansara et al. 2018 | Alvarado | 7 | 68.9 | 73.1 | 87.9 | 45.2 | 70 | 12.1 | NA |
| Patil et al. 2018 | Ripasa | 7.5 | 95.5 | 89.7 | 95 | 89 | NA | NA | 0.926 |

| Patil et al. 2018 | Alvarado | 7 | 81.1 | 87.2 | 81 | 87 | NA | NA | 0.841 |
| --- | --- | --- | --- | --- | --- | --- | --- | --- | --- |
| Chavan et al. 2018 | Ripasa | 7.5 | 90.8 | 100 | 100 | 10 | 90 | 0 | NA |
| Chavan et al. 2018 | Alvarado | 7 | 75.8 | 100 | 100 | 4 | 76 | 0 | NA |
| Abdelrhman et al. 2018 | Ripasa | 7.5 | 95.5 | 72.7 | 96.6 | 66.7 | 93 | NA | 0.950 |
| Abdelrhman et al. 2018 | Alvarado | 7 | 73 | 81.8 | 97 | 27.3 | 74 | NA | 0.740 |
| Abdelrhman et al. 2018 | Ripasa | 7.5 | 97.6 | 66.7 | 93 | 85.7 | 92 | NA | 0.870 |
| Abdelrhman et al. 2018 | Alvarado | 7 | 79.3 | 83.3 | 95.6 | 46.9 | 80 | NA | 0.860 |
| Pasumarthi et al. 2018 | Ripasa | 7.5 | 75 | 65 | 91.1 | 35.1 | 73.3 | NA | 0.810 |
| Pasumarthi et al. 2018 | Alvarado | 6 | 52.1 | 80 | 92.6 | 25.8 | 56.9 | NA | 0.771 |
| Elhosseiny et al. 2018 | Ripasa | 7.5 | 100 | 75 | 95.8 | 100 | 88 | 4.2 | NA |
| Elhosseiny et al. 2018 | Alvarado | 7 | 65.2 | 100 | 100 | 33.3 | 83 | 0 | NA |
| Nancharaiah et al. 2018 | Ripasa | 7.5 | 98.6 | 83.3 | 93.3 | 71.4 | NA | NA | 0.892 |
| Nancharaiah et al. 2018 | Alvarado | 7 | 76.4 | 66.7 | 89 | 10.5 | NA | NA | 0.757 |

| Arroyo-Rangel et al.  2017 | Ripasa | NA | 99 | 71 | 96 | 91 | NA | NA | 0.880 |
| --- | --- | --- | --- | --- | --- | --- | --- | --- | --- |
| Arroyo-Rangel et al.  2017 | Alvarado | NA | 91 | 64 | 94 | 60 | NA | NA | 0.800 |
| Rodrigues et al. 2017 | Ripasa | 7.5 | 93 | 31.6 | 86 | 50 | NA | NA | NA |
| Rodrigues et al. 2017 | Alvarado | 7 | 81.4 | 47.4 | 87.5 | 36 | NA | NA | NA |
| Karami et al. 2017 | Ripasa | 8 | 93.2 | 91.7 | 98.8 | 64.7 | NA | NA | 0.980 |
| Karami et al. 2017 | Alvarado | 7 | 78.4 | 100 | 100 | 38.7 | NA | NA | 0.910 |
| Chae et al. 2017 | Ripasa | 7.5 | 16.4 | 99.2 | 90.9 | 71.3 | 65.3 | NA | 0.650 |
| Chae et al. 2017 | Alvarado | 7 | 14.8 | 95.3 | 60 | 70.1 | 69.8 | NA | 0.700 |
| Regar et al. 2017 | Ripasa | 7.5 | 94.7 | 60 | 97.8 | 37.5 | 93 | 2.2 | NA |
| Regar et al. 2017 | Alvarado | 7 | 67.4 | 80 | 98.5 | 11.4 | 68 | 1.5 | NA |
| Subramani et al. | Ripasa | 7.5 | 98 | 80.4 | 84.4 | 97.4 | 89.6 | 15.5 | NA |
| Subramani et al. | Alvarado | 7 | 68 | 86.9 | 85 | 71.4 | 77.1 | 15 | NA |

| Golden et al. 2016 | Ripasa | 7.5 | 78 | 36 | 39 | 76 | NA | NA | 0.670 |
| --- | --- | --- | --- | --- | --- | --- | --- | --- | --- |
| Golden et al. 2016 | Alvarado | 7 | 61 | 74 | 53 | 79 | NA | NA | 0.720 |
| Muduli et al. 2016 | Ripasa | 7.5 | 97.3 | 75 | 89.9 | 92.3 | 90.5 | NA | NA |
| Muduli et al. 2016 | Alvarado | 7 | 68.5 | 84.4 | 90.1 | 54 | 73.3 | NA | NA |
| Sinnet et al. 2016 | Ripasa | 7.5 | 95.5 | 65 | 92.4 | 76.5 | 89.9 | 7.6 | 0.943 |
| Sinnet et al. 2016 | Alvarado | 7 | 65.2 | 90 | 96.7 | 36.7 | 69.7 | 3.3 | 0.862 |
| Liu et al. 2015 | Ripasa | 7.5 | 95.2 | 73.6 | NA | NA | 87.2 | NA | NA |
| Liu et al. 2015 | Alvarado | 7 | 63.1 | 80.9 | NA | NA | 69.7 | NA | NA |
| Srikantaiah et al. 2015 | Ripasa | 7.5 | 95.5 | 89.7 | 95 | 89 | NA | NA | 0.926 |
| Srikantaiah et al. 2015 | Alvarado | 7 | 81.1 | 87.2 | 81 | 87 | NA | NA | 0.841 |
| Verma et al. 2015 | Ripasa | 7.5 | 100 | 11.1 | 91.9 | 100 | 92 | 8.1 | NA |
| Verma et al. 2015 | Alvarado | 7 | 82.4 | 44.4 | 93.7 | 20 | 79 | 6.3 | NA |
| Walczak et al. 2015 | Ripasa | 7.5 | 88 | 9 | 68 | 20 | NA | NA | NA |
| Walczak et al. 2015 | Alvarado | 7 | 85 | 16 | 74 | 29 | NA | NA | NA |
| NaNjuNdaiah et al. 2014 | Ripasa | 7.5 | 96.2 | 90.5 | 98.9 | 73.1 | 96.2 | NA | 0.982 |
| NaNjuNdaiah et al. 2014 | Alvarado | 7 | 58.9 | 85.7 | 97.3 | 19.1 | 58.9 | NA | 0.849 |
| Erdem et al. 2013 | Ripasa | 7.5 | 100 | 28 | 75 | 100 | 77 | 25 | 0.857 |
| Erdem et al. 2013 | Alvarado | 7 | 82 | 75 | 88 | 66 | 80 | 12 | 0.818 |
| Erdem et al. 2013 | Ripasa | 10.2 | 83 | 75 | NA | NA | NA | NA | 0.857 |
| Erdem et al. 2013 | Alvarado | 6.5 | 82 | 75 | NA | NA | NA | NA | 0.818 |
| Alnjadat et al. 2013 | Ripasa | 7.5 | 93.2 | 61.8 | 92.2 | 64.9 | 91.5 | 7.8 | 0.914 |
| Alnjadat et al. 2013 | Alvarado | 7 | 73.7 | 68.6 | 92 | 34.8 | 74.3 | 8 | 0.743 |
| Chong et al.2011 | Ripasa | 7.5 | 98 | 81.3 | 85.3 | 97.4 | 91.8 | 14.7 | 0.918 |
| Chong et al.2011 | Alvarado | 7 | 68.3 | 87.9 | 86.2 | 71.4 | 86.5 | 13.8 | 0.865 |

[Click here to access/download;Figure;Fig 1.tiff](https://www.editorialmanager.com/pone/download.aspx?id=31598023&guid=3e1b0949-492d-4c1d-bd16-92ef32538ecf&scheme=1)

[Click here to access/download;Figure;Fig 2.TI](https://www.editorialmanager.com/pone/download.aspx?id=31598024&guid=802bf77b-aa67-46f9-bdbb-a2d2d253483d&scheme=1)

[Click here to access/download;Figure;Fig 3.TI](https://www.editorialmanager.com/pone/download.aspx?id=31598025&guid=bcc1ce8f-b71a-429b-91ad-8b02611a3956&scheme=1)

[Click here to access/download;Figure;Fig 4.TI](https://www.editorialmanager.com/pone/download.aspx?id=31598026&guid=6292cba0-ff94-4461-895a-b229c572fa2c&scheme=1)

Additional File 1

[Click here to access/download](https://www.editorialmanager.com/pone/download.aspx?id=31598036&guid=cce2bbe1-1768-4745-babf-8ae0fc425a1c&scheme=1)

[Supporting Information](https://www.editorialmanager.com/pone/download.aspx?id=31598036&guid=cce2bbe1-1768-4745-babf-8ae0fc425a1c&scheme=1)

[Additional file 1.docx](https://www.editorialmanager.com/pone/download.aspx?id=31598036&guid=cce2bbe1-1768-4745-babf-8ae0fc425a1c&scheme=1)

Additional File 2

[Click here to access/download](https://www.editorialmanager.com/pone/download.aspx?id=31598038&guid=67160ee4-0d66-4eb4-a46b-c77a5e5e2f34&scheme=1)

[Supporting Information](https://www.editorialmanager.com/pone/download.aspx?id=31598038&guid=67160ee4-0d66-4eb4-a46b-c77a5e5e2f34&scheme=1)

[Additional file 2.docx](https://www.editorialmanager.com/pone/download.aspx?id=31598038&guid=67160ee4-0d66-4eb4-a46b-c77a5e5e2f34&scheme=1)
